# A phenomenological study on the traces of obstetric violence in women’s birth experiences

**DOI:** 10.1186/s12884-025-08426-x

**Published:** 2025-11-14

**Authors:** Kubra Karabulut, Aysegul Unutkan

**Affiliations:** 1 Kutahya Health Sciences University Training and Research Hospital, Kutahya, Türkiye; 2 Department of Midwifery, Kutahya Health Sciences University Health Sciences Faculty, PO Box 43700, Kutahya, Türkiye

**Keywords:** Birth experience, Labor, Obstetric violence, Mistreatment, Abuse, Disrespect, Midwifery

## Abstract

**Aim:**

In this study, we aimed to examine women’s birth experiences in the context of obstetric violence.

**Design & method:**

This is a qualitative study examining women’s birth experiences phenomenologically. The research data were collected from 25 pregnant women who visited the Training and Research Hospital Electronic Fetal Monitorization outpatient clinic who were in the last trimester of pregnancy and who had previously given birth vaginally. Descriptive information form and semi-structured interview form were used for data collection. A thematic content analysis approach was used to analyze the data. Thematic content analysis was performed based on the obstetric severity classification proposed by Bohren et al. and Bowser and Hill. MAXQDA 20 software program was used to code the data.

**Results:**

Women’s birth experiences were grouped under three themes linked to obstetric violence: dignified care, loss of autonomy, abandonment in care. Under these themes, eight categories were created: physical violence, verbal violence, deprivation of privacy, it was as if I did not exist, the medicalization of childbirth, intimidation/threatening with bad consequences to get what they wanted, midwives were like robots, I felt forgotten.

**Conclusion:**

It was determined that women were exposed to different types of obstetric violence during the delivery process. In order to prevent obstetric violence, it is important to increase the awareness of midwives, obstetricians, and care recipients on the issue and to solve structural problems such as lack of resources.

## Introduction

Many studies in recent years have drawn attention to the disrespectful treatment of women in childbirth [1–5]. In 2014, the World Health Organization (WHO) [2] defined attitudes such as physical harassment, verbal harassment, humiliation, coercive or non-consensual treatment, disregard for privacy, refusal to give painkillers, refusal to admit to the health institution, neglect during childbirth, endangering life, and detaining women and newborns in the facility as disrespectful and aggressive behavior in health institutions. The WHO does not directly use the term “obstetric violence” (OV). There is no consensus among researchers on terminology and definition. Chervenak et al., [6] argue that the term “obstetric violence” is a misnomer and that “obstetric maltreatment” should be used instead. However, some authors argue that labelling obstetric maltreatment downplays violence during childbirth and therefore should be considered an actual act of violence [7, 8]. The definition of “obstetric violence” was first legalized in Venezuela in 2007 with the Organic Law on the Right of Women to a Life Free of Violence. According to this law, obstetric violence is defined as “*the appropriation of the bodily and reproductive processes of women by health personnel*,* which is expressed as dehumanized treatment and an abuse of medication*,* and the transformation of the natural processes into pathological ones*,* bringing with it the loss of autonomy and the ability to decide freely about their bodies and sexuality*,* negatively impacting the quality of life of women”* [7].

Freedman and Kruk [9] define obstetric violence as a specific form of gender-based violence targeting women in healthcare settings during pregnancy, childbirth, and the postpartum period, violating human rights and evidence-based medical care. Chadwick [10] defines obstetric violence as violence resulting from power relations between healthcare professionals and patients. According to the United Nations, obstetric violence stems from structural inequality, discrimination, patriarchal systems, and a lack of education, as well as a lack of respect for gender equality and human rights [5, 10, 11]. The prevalence of OV worldwide varies between 18.3% and 75% [1, 4]. Studies conducted in Türkiye report that approximately 75% of women are exposed to some type of OV during birth [12, 13].

Studies have reported that women worldwide are exposed to different types of OV in childbirth [1, 2, 4, 5]. Bohren et al. [1] defined these types of violence as physical, sexual, and verbal violence, stigmatization and discrimination, failure to meet professional standards of care, poor rapport between women and providers, and restrictions in health system conditions. Other studies have reported that OV is associated with variables such as age, ethnicity, education, and socio-economic level [2, 14]. In this study, OV types were examined by taking into account Bohren’s classification. When the causes of OV are examined, although there are many reasons, the most common reasons are gender, ignorance and neglect of OV, organizational problems, power relations, racism, and the medicalization of childbirth [15].

One of the problems that makes OV important is the negative consequences it causes in terms of maternal and infant health and family relationships. In women who had negative birth experiences, postpartum mood disorders, personality changes, birth trauma and postpartum posttraumatic stress disorder (PTSD), deterioration of the bond with the newborn, negative effects on family relationships, impaired sexual functioning, and not wanting another child were observed [16, 17]. As a result, the OV experienced by women during childbirth can cause physical and psychological resentment [18]. The search for the respectful birth experience that women deserve in births has spread to other countries with the ‘Humanization of Birth’ movement, starting in Latin American countries under the leadership of midwives [19, 20].

Although the importance of OV has been recognized in different countries around the world, this topic is very new for Türkiye. There are a limited number of studies on the subject in Türkiye [11, 12, 21–23]. Further qualitative analysis is necessary to find out more about women’s experiences of potentially violent treatment during childbirth in Türkiye. Although the term “OV” is increasingly used in academic and advocacy contexts, it currently lacks a standardized legal definition not only in Türkiye but also at the global level. Therefore, developing strategies and interventions to prevent OV is important. Therefore, this study utilized women’s narratives about their birth experiences to understand how women perceive OV and what its short- and long-term consequences are. Making OV visible is the first step towards actions that can be taken to improve women’s emotional and physical safety in labor and the quality of care they receive and to ensure that the dialogue between women and health professionals is based on mutual respect, trust, and understanding. Accordingly, this study seeks to clarify the nature, types, and perceived consequences of obstetric violence as reflected in women’s narratives of their birth experiences.

## Material and method

### Aim

This study aimed to examine women’s birth experiences in the context of OV.

### Design

This is a qualitative study conducted to examine women’s birth experiences in the context of OV phenomenologically. In this study, hermeneutic phenomenology based on the Heideggerian and Gadamerian approach was used to gain an in-depth understanding of women’s experiences of obstetric violence. The Heideggerian approach, which aims to reveal the existential meanings individuals attach to their experiences rather than simply describing them, provides a suitable framework for analyzing the impact of obstetric violence on women’s perception of their bodies, identities, and autonomy. Gadamer’s hermeneutic framework, however, stresses that meaning arises from the dialogue between the participant and the researcher and the fusion of their horizons. In this direction, the data collection process was carried out through open-ended interviews that delved deeply into the experiences women shared in their contexts; the analysis process focused on interpreting multi-layered meanings with continuous interaction with the text and awareness of pre-understanding [24]. The Consolidated Criteria for Reporting Qualitative Research (COREQ) was used as a guide throughout all stages of the research [25].

### Study population and sample

The study population consisted of multiparous pregnant women in their last trimester. Twenty-five women who matched the inclusion criteria and arrived at the training and research hospital’s Electronic Fetal Monitorization (EFM) outpatient clinic between June 6 and December 30, 2022, made up the study sample. EFM is a test performed to assess fetal well-being in the last trimester. A midwife uses a cardiotocography device at the EFM outpatient clinic to evaluate and record pregnant women in their last trimester. The study took place in a tertiary healthcare facility that has a capacity of 950 beds. The number of births at the hospital varies between 100 and 130 per month. An average of 500 pregnant women present to the EFM clinic monthly.

The study sample was determined by the criterion sampling method, one of the purposive sampling methods. The inclusion criteria were having previously given birth vaginally, being more than five years since the last birth, being in the last trimester of pregnancy, having no history of stillbirth, volunteering to participate in the study, and being able to speak and understand Turkish. The first researcher works in the EFM outpatient clinic and could not leave the work area between 8:00 and 17:00. Pregnant women were selected as participants due to ease of access to the study sample. The criterion of a maximum of five years since the previous birth is a methodologically critical threshold for preserving the vivid, detailed, and emotional layers of meaning associated with the birth memory. As time passes, memory reconsolidation and forgetfulness tendencies can blur the meaning of the experience. Therefore, women who had a maximum of five years since their previous birth were included. Exclusion criteria were having difficulty in speaking and understanding our language and having a mental disorder that would make it difficult to communicate.

In qualitative research designs, sample size is determined in line with the research question and purpose. Interviews should be continued until no new information/opinions emerge. Interviews were terminated when the concepts that could be the answer to the research question started to repeat (saturation point). Four themes were used as criteria for data saturation: undignified care, abandonment in care, deprivation of privacy, and loss of autonomy.

### Data collection

We sought to answer the question: ‘Which types of OV do women’s narratives about their past birth experiences?’ and ‘How were women affected by these experiences?’ The data of the study were collected through in-depth interviews. The first researcher conducted the interviews. Introductory information form and semi-structured interview form were used to collect the data.

### Semi-structured interview form

Before preparing the interview questions, national and international literature on the research topic was reviewed, and qualitative and quantitative studies on the subject were utilized [1, 26, 27]. Five open-ended questions were created aiming to reveal the traces of OV experienced by women in their birth experiences. The following questions refer to the interview protocol.

1. Please share your previous birthing experience?
2. What made you feel happy during your birth?
3. What were the things that made you feel sad during your birth?
4. If you could go back to the moment of birth, what would you change?
5. If you had to compare your birth to something, what would it be?

In addition, probing questions such as “Is there anything else you would like to say about this issue?”, “What did you feel in this situation?”, and “What do you believe concerning this issue?” were asked to deepen the answers given by the participant during the interview. To assess the feasibility of the instruments to be used during the study’s preparation and data collection process, the interview questions were presented to researchers experienced in qualitative research for review. The final version was finalized after necessary revisions were made based on their suggestions. Two pregnant women, not included in the sample, were interviewed using the research questions before beginning the data collection process. These interviews determined the feasibility of the data collection instruments.

### Rigour and reflexivity

The study used an individual in-depth interview technique for the data collection. The researcher introduced herself to the pregnant women who applied to the EFM outpatient clinic and explained the purpose of the research, how it would be conducted, that voice recordings would be taken, and that they would not be harmed for participating in the research. Appointments were made with pregnant women who agreed to participate in the study. The participants did not know the researchers beforehand. Interviews were conducted in a room where the participant and the researcher could sit face to face, the interview would not be interrupted, the pregnant woman could lie down or sit down during the interview if she wished, and there was good ventilation and lighting. Before starting the interview, the researcher explained to the researcher could take notes, that her name would not be deciphered anywhere in line with the information she provided, and that she would be respected if she wanted to terminate the interview. Using a semi-structured interview form during the interviews facilitated the realization of the interviews in a logical sequence. In-depth interviews lasting approximately 60 min were conducted with each pregnant woman. At the same time, short observation notes about the pregnant women’s body movements and voice tones were kept during the interview. Before the interviews were finalized, it was confirmed whether the participants had anything they wanted to add or ask, and the interview was terminated. There were not any repeated or interrupted interviews. When the participants later reapplied to the EFM outpatient clinic, participant confirmation was obtained regarding the deciphered data. After the research was completed, the data was anonymized and stored in a cloud account, accessible only to the authors. In the article, the names of the participants are coded with numbers and given in parentheses with their ages at the end of the quotations (Participant number, age).

We established an ethical support mechanism for participants who were likely to experience emotional distress after the interviews. A brief debriefing was conducted after the interviews, participants’ emotional states were monitored, and a plan was made to refer them to the university’s psychological counseling unit or local mental health services if necessary. Throughout the research process, the researcher prioritized the psychological safety of the participants, ensuring that no participant was left in a negative emotional state. Furthermore, participants were provided with the researcher’s contact information after the interviews and they were informed that they could contact the researcher if they needed support in the future. However, no participants requested support.

### Data analysis

The data were analyzed by the researchers. First researcher: The researcher was involved in the research process after receiving 16 h of training in qualitative research. The researcher, who worked as a midwife in the delivery room for four years and in the EFM outpatient clinic for the last two, was able to interpret participant statements richly and contextually thanks to her profound field knowledge of clinical processes and power dynamics in the birth environment. Second researcher: with a doctorate in obstetrics and gynecology nursing, thirteen years of academic experience in midwifery, and publications in the field of qualitative research, she analyzed the data with methodological rigour and theoretical depth.

The data were analyzed using the thematic content analysis method. Thematic content analysis was conducted based on the six steps suggested by Clarke and Braun [28] (Table 1). The coding framework for the analysis was based on the types of maltreatment defined by both Bohren et al. [1] and Bowser and Hill [29]. All themes overlapped in content with these classifications.

**Table 1:** Thematic content analysis steps of the study

Step 1: Familiarization with the data	Interviews with the participants were recorded with a digital voice recorder. The interviews were listened to one by one on the same day, transcribed, and computerized. In order to increase impartiality, internal control was ensured by listening to the interview recording after each interview. The data of 25 participants were transcribed, resulting in a 152-page document. Then, the researcher listened to it again and organized it according to the interview transcription rules. The data and observation notes were read over and over again by two researchers to ensure data familiarity.
Step 2: Generation of initial codes	In this step, the researchers first reviewed the data for potential items of interest, units of meaning, and connections and noted preliminary ideas. Then, they defined the coding framework and coded all the data individually. MAXQDA 20 software program was used to code the data.
Step 3: Searching for themes	First, the coded and compiled data excerpts were analyzed in depth, and possible themes were noted. Clarke and Braun (2017) described individual codes as bricks and tiles and themes as walls and roofs when the whole analysis is considered a house. At this stage, the researchers reached draft themes by analyzing, combining, comparing, and even mapping the relationship between the codes.
Step 4: Review of themes	In this step, the authors first reviewed the data coded under each theme. This process examined whether the themes had sufficient supporting data and whether the data under the themes were consistent. As a result of these reviews, some themes were combined, and some were removed. It was ensured that the final thematic map adequately and accurately represented all the data.
Step 5: Identifying and naming themes	In this step, themes and categories were named. It was ensured that the themes and categories explained the underlying content and served the whole topic. The themes and categories were first named from the coded excerpts. Then, in order for the themes to reflect the content, they were named by adding the theoretical framework explained next to the quotations. The two authors first agreed upon the thematic map and theme names, and then the themes were finalized by getting the opinion of an expert experienced in qualitative research.
Step 6: Production of the report	Themes and coding were explained, supported by representative data citations. The findings were interpreted by comparing them with the literature. The names of the participants were coded in the text. The codes and ages of the participants were indicated next to the representative quotations.

### Ethical aspects of research

Approval was obtained from the Kütahya Health Sciences University Non-interventional Research Ethics Committee (date: 06.04.2022 number: 2022/04–16) before starting the study. Written informed consent was obtained from pregnant women who agreed to participate in the study. It was stated that their identity information would be kept confidential, audio recordings and documents would not be shared, and they could terminate the interview at any time. The study adhered to the current Declaration of Helsinki.

## Results

### Participants’ characteristics

The mean age of the pregnant women who participated in the study was 28.6 years. Eight participants were primary school graduates, 14 were high school graduates, and three were university graduates. Two pregnant women who participated in the study were employed in a paid job, and 23 were housewives. All of the participants were multiparous pregnant women in the last trimester, and their most recent delivery was vaginal. The descriptive characteristics of the participants are presented in Table 2.

**Table 2:** Characteristics of participants

Participant	Age	Education Level	Job	Number of Pregnancy
1	27	High School	Housewife	2
2	26	High School	Housewife	2
3	28	Primary School	Housewife	2
4	36	High School	Housewife	4
5	35	High School	Housewife	2
6	35	High School	Housewife	2
7	23	High School	Housewife	2
8	25	High School	Housewife	2
9	29	Universty	Judge	2
10	28	High School	Housewife	2
11	25	Universty	Housewife	2
12	37	Primary School	Housewife	3
13	34	High School	Housewife	2
14	30	Primary School	Housewife	3
15	30	High School	Housewife	2
16	26	High School	Housewife	3
17	30	Primary School	Housewife	2
18	23	High School	Housewife	2
19	25	Primary School	Housewife	4
20	32	High School	Housewife	4
21	29	Associate degree	Pre-school teacher	2
22	21	High School	Housewife	2
23	27	Primary School	Housewife	2
24	32	Primary School	Housewife	4
25	32	Primary School	Housewife	3

### Women’s birth experiences

After transcribing the interviews conducted for the study, three main themes and eight categories under these main themes were identified (Table 3).

**Table 3:** Themes and categories

THEMES	CATEGORIES
Dishonorable Care	Physical Violence
	Verbal Violence
	Deprivation of Privacy
Loss of Autonomy	‘It was as if I did not exist.’
	Medicalization of Childbirth
	Intimidation/Threatening with Bad Consequences to Get What They Want
Abandonment in Care	‘Midwives were as if they were robots.’
	‘I felt like I was forgotten.’

#### Dishonorable care

Under the theme of dishonorable care, three categories were identified: physical violence, verbal violence, and deprivation of privacy.

##### Category 1: physical violence

Participants stated that they were tied up at birth and that they were uncomfortable with this situation and even felt like a sacrificial lamb or a criminal. It was also determined that the participants suffered physical pain due to the kristeller maneuver (the Kristeller maneuver is the process of applying pressure to the uterine fundus manually or with the forearm to accelerate the delivery of the baby during the second stage of labor) at birth, that they were in pain for days after birth due to the pressure applied, and that the procedure was performed without information. In addition, the participants stated that episiotomy repair was performed without anesthesia or that they felt that the anesthesia was inadequate.

*“They tied my hands and feet so that I would not kick during labor. Then*,* when I thought about it*,* I felt bad*,* like a sacrificial lamb*,* and at that moment*,* I said okay with the pain.“(P17*,* 30)*.

##### Category 2: verbal violence

Participants reported that birth workers were harsh and rude to them/their families, scolding them with commands such as ‘do not shout,’ ‘push,’ ‘do not move,’ scolding them for not pushing or being non-compliant, raising their voices at them, and insulting them because of their physical appearance or fussiness about childbirth. In addition, women reported hearing insensitive and sarcastic remarks such as ‘You will not be able to give birth at this rate,’ ‘If you are pregnant, you will bear the consequences,’ and ‘What is there to shout about’ instead of emotional support and encouraging.

“*They said that if you are pregnant*,* you will bear the consequences. They did not treat us like human beings. We were treated like animals. They were very harsh.” (P16*,* 26)*.

*“They tried many times to open an intravenous line*,* but it didn’t work*,* and I couldn’t stand it with the pain of labor. I reacted slightly*,* and he laughed*,* saying*,* “Are you the only one giving birth?” (P21*,* 29)*.

##### Category 3: deprivation of privacy

Participants reported that their privacy was not protected during childbirth. Women reported that they felt uncomfortable and embarrassed when they were being watched by too many students during labor and delivery. It was also found that women were examined too often and by different people without their privacy being protected. In addition, pregnant women were laid side by side in the same room; there was only a curtain between the beds, and they heard the shouting of other women and were negatively affected by this.

*“I am normally shy; privacy was not paid attention to. It is crowded*,* and there are one or two*,* let’s say three midwives*,* but there are many of them gathered*,* men and so on*,* all looking simultaneously; it makes you feel embarrassed. When you are being examined*,* the doors are open*,* and other patients are looking at you; I tell them to close the door*,* and they push it with their hand*,* but the door opens again. There were many people*,* students. It was very crowded. I have never been as embarrassed as I was during childbirth. No attention was paid to privacy.” (P25*,* 32)*.

#### Loss of autonomy

‘It is my body but not my decision’: Under the theme of loss of autonomy, three categories were identified: ‘it was as if I did not exist,’ medicalization of childbirth, and intimidation/threats of bad consequences to get what they wanted. In this theme, there are overlapping statements regarding the lack of consent in the first two categories. We decided to present the two themes separately because the first theme focuses on the woman’s subjective experience and the feeling of being ignored, while the second highlights the institutional and structural aspects of maternity care. While both emphasize the loss of autonomy, we believe presenting them separately will enhance understanding of the multilayered nature of the phenomenon by making both individual perceptions and systemic practices visible. Additionally, threats of negative consequences can technically be classified as a subtype of verbal violence. However, this category was considered separately because threatening rhetoric is not merely a form of hurtful or degrading communication but rather a systematic tool used to pressure women, manipulate their behavior, and exert control. Considering threats solely within the scope of “verbal violence” can obscure the power relations behind this behavior, attempts to establish authority, and the structural dimensions that undermine women’s autonomy. Therefore, we separate this category from verbal violence.

##### Category 1: ‘It was as if I did not exist.’

Women who participated in our study stated that they were not informed about the interventions performed during the birth process, consent was not obtained, and that they learned about the interventions after they were performed. The participants reported that they would not have allowed the interventions if they had been asked for permission before the procedures.

*“I was stitched from below*,* but no one said anything to me. They talked about something among themselves*,* but no one said anything to me; it was like I did not exist.” (P1*,* 27)*.

*“Something was done during the birthing process. No one asked me anything. Everything was done by force; I felt like I was a mechanical person. It was my body*,* but it wasn’t my decision. It was as if I wasn’t there.” (P16*,* 26)*.

##### Category 2: medicalization of childbirth

Women who participated in the study were asked to stay in bed during the childbirth, and were constantly connected to the EFM despite the lack of risk. It was understood that they were not allowed to walk, their freedom of movement was taken away, and they could not be active in the process. Women stated that they had great difficulty due to the prohibition of oral intake during the childbirth. Women stated that induction was performed after admission to the hospital with inadequate information and without consent. It was understood that all participants underwent routine episiotomy and were not adequately informed beforehand. Women stated that they had difficulty breathing and moving after the kristeller maneuver because they were caught unprepared and that they would not have given consent if they had been informed beforehand.

*“I wanted to get up*,* but they would not let me; the pain hit me directly in my back. I did not feel it when I was standing*,* but when I was lying down*,* the pain hit my back directly; I felt it more. I said I wanted to walk*,* and they said no. I had to stay connected to the EFM.” (P1*,* 27)*.

*“It was forbidden to eat or drink anything during the labor process. I wish I could have water; it would be wonderful.” (P22*,* 21).*

##### Category 3: intimidation/threatening with bad consequences to get what they want

The participants in our study reported feeling intimidated by midwives and obstetricians associated with adverse birth outcomes, indicating that they felt compelled to acquiesce to requests and frequently faced threats of being ignored for care. They stated that they often heard phrases such as ‘if you close your legs, your baby will get stuck,’ ‘if you do not push, your baby will die,’ and ‘if you do this, I will not deliver you’ from midwives during labor. When they shouted because of the pain they felt during episiotomy repair, they were told that they had to endure the pain; otherwise, there was a risk of death. The women stated that over time, they forgot the pain they experienced during labor, but they could not forget the fear that the words made them feel.

*“When the doctor was stitching her up after the birth because I told him that I was in much pain*,* he said*,* ‘I will not stitch this woman up*,* let her stay crooked*,* her husband will not like it*,*’ and left the stitching unfinished. The midwives completed the stitching.” (P5*,* 35).*

*“I closed my legs while lying on the delivery table. The midwife said something like*,* ‘If you close your legs*,* the baby’s head will become stuck. I could have accidentally crushed my baby’s head and feared that I might kill him. I was terrified I might hurt him. I’ll never forget that fear.” (P 21*,* 29)*.

#### Abandonment in care

Under the theme of abandonment in care, two categories were identified: ' Midwives were like robots’ and ‘I felt forgotten.’ Both categories under this theme revolve around a lack of support. There are overlapping statements, but the “Midwives were like robots” category points to the reduction of care to a technical routine and the insensitivity of professional care routines. The second category highlights the lack of social and emotional support, emphasizing the feelings of loneliness and abandonment experienced by women.

##### Category 1: ‘Midwives were as if they were robots.’

In our study, women stated that health professionals acted business-oriented during the birth process and only saw the patients as tasks that needed to be done. Some participants made the analogy that ‘They were like robots’ for healthcare professionals. From the participant statements, it was understood that women did not receive the support they expected as a result of health professionals being only interested in their duties and not communicating. In addition, women reported that midwives ignored their wishes and ideas, avoided explanations about their care, and rushed or did not answer their questions. Women also reported that their concerns were ignored. All of the women expressed that they needed the support of a caring partner or a close person to provide compassion and care during their births but that they expected this support from health professionals as companion support was prohibited in the hospital, and their expectations were unmet.

*“Everyone did their own thing and left; no one took care of me. They did not say a bad word; the same midwives came and went*,* and nothing positive was said; they were like robots*,* like ‘come and go*,* do it.’ It was like*,* “Let’s deliver birth*,* let’s take the patient out*,*” and that was it. They simply performed their duties*,* returned to their rooms*,* and sat down. There was no human relationship*,* like a robot. I gave birth in the hospital*,* and that was it.” (K8 25)*.

##### Category 2: ‘I felt like I was forgotten.’

The women who participated in the study stated that they were left alone during labor. They stated that midwives work in a work-oriented manner; they do the procedures and leave the room, they do not come when they are called, and even if they come, they hear the words “Did you call for this?“. They reported that health professionals were only with them when their babies were born; otherwise, they were left alone. Women also stated that they wanted to spend the birth process with their husbands or family members but were not allowed to. Therefore, they described the birth process as ‘all alone’ and ‘lonely.’

*“There is no one; I am lying in bed connected to the EFM. I call out*,* but they do not hear me. They go to their rooms and do not come. I fainted here*,* I woke up*,* and I vomited. You are bedridden; you cannot get up. It was a very difficult process. Then I struck the bed’s iron frame with the IV pole*,* and they responded. I felt very bad; no one would care if I stayed or died; it was like I was forgotten.” (P5*,* 35)*.

*“I didn’t receive the support I expected from the midwives. They focused on medical procedures*,* vaginal examinations*,* and EFM. There was no psychological support.” (P9*,* 29)*.

## Discussion

This study aims to examine the previous birth experiences of pregnant women in the context of OV and to offer recommendations for enhancing maternity care services accordingly. In contrast to several international studies, research on OV in Turkey is scarce, and the phenomenon frequently lacks enough awareness in both academic discourse and health policy. This study provides a distinctive addition by contextualizing the analysis within Turkey, recording women’s lived experiences, and examining the cultural and systemic processes influencing maternity care in the country. The study aims to enhance awareness of OV as a vital public health and human rights concern, while addressing a notable deficiency in the local literature. The findings seek to educate policy-makers and healthcare providers on measures to enhance respectful maternity care and to avert behaviors that could compromise women’s autonomy and well-being during birthing. In this section, the findings obtained in line with the objectives of the study are discussed and interpreted in a certain order in the light of themes and coding.

In many parts of the world, women are exposed to many forms of OV during delivery. Women describe this situation as *‘leaving their dignity at the door’* [30]. Similarly, in Rodriguez et al.‘s [26] study, one of the participants expressed the inhumane approach she encountered in childbirth with the words, “*We were treated like cattle.”* In the current study, in line with the literature, women expressed the bad behaviors they were exposed to with the sentence *‘We were treated like animals’* when describing their birth experiences. Behaviors that are unworthy of human dignity are often described with this metaphor. This statement draws attention to the inhuman treatment in childbirth from a woman’s perspective. Although women express these mistreatments so strikingly, on the other hand, they accept these behaviors as the norm of childbirth.

Women in the current study reported experiencing physical violence such as frequent vaginal examinations and crude Kristeller maneuvers. In other studies supporting our study findings, it was reported that women were exposed to physical violence [26, 31]. Unfortunately, such practices are perceived as necessary interventions in situations considered ‘difficult’ by birth workers, which contributes to the banalization and invisibility of violence. On the other hand, in the present study, women were frequently scolded, yelled at, and humiliated during labor. Similarly, other studies have reported that when women raised their voices during labor pain, they were insulted [27, 32]. Moral and prejudiced jokes are perceived as a form of humor by birth professionals [31, 33, 34]. However, the use of embarrassing remarks and irony in childbirth can damage women’s trust in obstetric professionals and the health care system. At this point, it is critical that health professionals first define their behaviors as acts of violence and raise awareness about this issue, subsequently, planning and implementing regular in-service training to remind them of professional ethical principles to strengthen the understanding of respectful care during the birth process is considered valuable in terms of improving the quality of care.

In the current study, most participants stated that their privacy was not taken into consideration. In another study, women described being left uncovered during childbirth and being treated carelessly during forced and frequent vaginal examinations with narratives similar to those of rape victims. In both cases, the language used by victims included expressions such as ‘being stripped,’ ‘tied up,’ ‘forced exposure,’ ‘display of genitals,’ ‘rendered powerless,’ and ‘chopped up like a piece of meat’ [35]. On the other hand, Tesser et al. [36] reported that women lay in labor without a screen. In order to increase the skills of students in vocational training processes, vaginal examinations are performed repeatedly by different people without paying attention to the privacy and consent of pregnant women, physical inadequacies of the facilities and delivery in ward order embarrass and disturb women. Diniz et al.’s [37] study emphasizes that women are treated as ‘training materials’, forgetting that they are human beings. This situation draws attention to the necessity of structuring education processes in a way that does not turn women into objects of education and respects their human rights.

Women who participated in the study stated that they did not have a say in their birth and that they felt as if they were watching their birth from the outside. Similarly, in Reyes-Foster’s [38] study, women stated that they did not feel included in the birth processes, were treated as if they did not exist by health professionals, their autonomy was taken away from them and left in a corner, their voices were not heard, and they watched decisions about them from afar like strangers. Claire Wendland [39] explained this loss of autonomy experienced by mothers at birth with the words ‘disappearance’. The word ‘disappearance’ describes a kind of exile of mothers from their own experiences. On the other hand, when the perspective of health professionals is examined, it is understood that they argue that they can make the best decision on behalf of women due to their knowledge and experience and that they can perform interventions without consent and consent because their duty is to save the life of the mother and the baby [5, 40]. In birth settings, sometimes decisions have to be made quickly, and there are at least two lives to consider. In such situations, respectful communication between the birthing mother and the midwife can contribute to women feeling involved in their birth.

Many women hospitalized for childbirth are subjected to routine but non-evidence-based practices [16, 19, 27]. In the current study, it was determined that women were deprived of their freedom of movement during the birth process due to continuous EFM or, for no reason, they were starved during labor, and almost all of them were subjected to episiotomy. The fact that physicians define pregnancy/birth as a “disease” causes them to resort to medical tools to manage the process. This leads to a medical approach that ignores women’s control over their bodies [6]. Women are becoming objects rather than subjects of childbirth due to the medicalization of the birth process much more than it should be [1, 38]. What is important in childbirth is not that the baby is born vaginally but that the choices of women in labor are respected and their voices are heard [38]. As emphasized in the Lancet Maternal Health series, interventions can be reduced with continuous support and midwife-led care in labor [41]. Accordingly, it is valuable to invest in increasing the competence and empowerment of midwives to reduce this type of OV.

The recently published new midwifery regulation have increased midwives’ autonomy, allowing them to assume a more active and independent role in the birth process [42]. In Turkiye, the medical responsibility for birth, including low-risk births, was largely delegated to physicians. This arrangement is believed to have led to physicians resorting to excessive interventions to expedite labor due to their heavy workload. Midwives’ obligation to be accountable to physicians for every birth may have limited their independence in decision-making, leading to a loss of motivation and a reluctance to provide compassionate care. However, by strengthening midwives’ professional autonomy in birth, the new regulation pave the way for woman-centered and respectful care and facilitate a transformation that could contribute to a reduction in interventionist practices.

Recent studies show that midwifery care models directly contribute to the prevention of obstetric violence by strengthening women’s autonomy and right to respect [20, 43]. By adopting a non-invasive approach, midwifery care helps reduce procedures performed outside of medical necessity, which can both increase birth safety and prevent traumatic experiences [44]. Therefore, empowering midwives and actively engaging them in the birth process is strategically important for both reducing obstetric violence and promoting a more humane birth practice [43]. Future studies could consider examining the impact of this investment in midwives on birth outcomes, quality of care, and women’s birth experiences in Türkiye as an important area of research.

Midwives have been described as ‘difficult’ patients, women who are uncooperative in labor, cover their feet, do not listen to the staff, cannot push effectively, disrupt the work of the staff, are disorganized, do not understand what the midwives are saying due to their level of education, ‘lose their mind’ (midwives use this term) due to the physiology of labor, are rude and refuse examination [30]. In such cases, health professionals may act more harshly, threaten, or raise their voices to force the patient to cooperate [31, 45]. They state that they do these behaviors to assist delivery or protect mother-infant health [31, 46, 47]. In line with the literature, all women in the present study were intimidated by health professionals during the birth process, fearing that they would harm their babies if they did not follow the commands. On the other hand, in studies conducted with midwives, midwives stated that they had to scare the mother in order to ensure her compliance in labor [46]. It is an ethical problem that midwives do not see such actions as violence. This contributes to the poor perception and perpetuation of the OV problem [45, 46]. Therefore, raising midwives’ awareness of this issue and training them on non-violent birth strategies seems important. On the other hand, midwives are under much pressure for successful births and are held responsible for negative birth outcomes. Their fear of things going wrong at birth and having to defend themselves in this regard may cause them to use abuse and force at birth. In this context, strategies to prevent OV should not only be limited to individual awareness and education but also require the development of structural support mechanisms at the system level (e.g., legal protection, team-based responsibility sharing, and professional support networks).

Women expect compassionate and understanding care from midwives during the difficult birth [48]. However, in the current study, women stated that midwives behaved indifferently and worked emotionlessly like robots. Similarly, in many studies, women complained that they did not receive adequate support during childbirth, were left alone, midwives did their work and left their rooms immediately, did not communicate, did not answer their questions, or passed them off with short and hasty answers [1, 31, 49]. In a study conducted with midwives, it is noteworthy that midwives’ perceptions of supportive care consisted of the health of the mother and newborn at birth and the provision of privacy [46]. It is important to underline that OV does not only consist of intentional mistreatment, insensitive care and inadequacies in birth environments can also expose women to mistreatment [35].

When it comes to maltreatment and insensitive care during childbirth, blaming birth workers, especially midwives, may seem like an easy option, but it does not offer a realistic approach to the problem. As mentioned earlier, midwives feel under much pressure to protect the health of the mother and baby at birth. Therefore, they experience stress and burnout [2]. A study of midwives and nurses reported that staff shortages, heavy workloads, poor infrastructure, and bottlenecks in labor and delivery can lead to the mistreatment of patients [50]. Our findings suggest that today’s midwives must balance their medical and humanitarian responsibilities during childbirth. Unfortunately, working conditions in maternity units do not allow the midwife to help the woman during her busy schedule. Insufficient financial resources for caring for women in labor and misuse of resources have led to this situation [51]. In order to prevent OV, it is important to look at the issue from a multidimensional perspective and address its structural dynamics rather than placing the blame solely on care providers [17, 52].

One of the most common complaints of women during childbirth is being left alone during labor. Women whose relatives were not allowed to attend the birth (usually citing the physical conditions of the hospital) stated that they felt abandoned [1, 31, 46, 47, 53]. In line with the literature, women in the current study stated that health professionals did not pay enough attention to them during the birth process and that they felt ‘*forgotten*’ in the delivery room. Similarly, in the study of Aguiar et al. [31] one of the participants stated that she felt unsupported in labor with the statement, “.*I felt like I was not being heard*.” The data collection process for the current study took place after the pandemic, so it is important to consider that COVID-19-related visitor restrictions and physical distancing measures may have impacted women’s birth experiences and their perceptions of obstetric violence. However, it’s known that in Türkiye, relatives were not allowed into births in previous years, except in mother-baby-friendly hospitals and some private hospitals. The strengthening of the mother-baby-friendly hospital policy over the past year has increased support for companions. We hope these developments will alleviate women’s loneliness.

### Limitations

Including women who gave birth within the last five years is an important limitation, as it may make it difficult to recall events. Although the qualitative nature of the study does not allow for generalization, it provides rich data showing that attention should be paid to the components of OV at the national level. Additionally, because this study’s sample is limited to women attending EFM outpatient clinics, the participants’ healthcare utilization characteristics and risk profiles may differ from the general pregnant population. Furthermore, the predominance of housewives in the sample may have led to narratives about birth experiences being particularly focused on socioeconomic status, thus relatively underrepresenting the lives of working-class women. This limitation constrains the findings’ generalizability, potentially overemphasizing some themes while overlooking others. Therefore, the results should be viewed as context-sensitive qualitative interpretations that do not assert prevalence, and one should also consider the impact of participant profiles and institutional contexts on the analysis. Despite all limitations, it is thought that the results of the study will add value to the literature.

## Conclusion

This study explored the OV experiences of women who gave birth vaginally. The results show that women are exposed to different types of OV. This study reveals that OV is an important problem with devastating consequences in our country. Multifaceted strategies need to be developed to prevent this type of violence. First of all, raising awareness of OV among birth workers and administrators of health institutions is valuable in drawing attention to acts of violence. On the other hand, there is an important need to strengthen the ethical values of birth workers, starting from their education. In addition, finding solutions to the problems stemming from the health system, physical deficiencies of health institutions, lack of personnel, and excessive workload is necessary for a holistic approach. Additionally, we believe that implementing curriculum reforms that include topics such as respectful birth, human rights, and effective communication in midwifery and medical education programs is a priority. Furthermore, establishing oversight mechanisms to monitor patient rights violations, improving the physical conditions of birth environments that respect privacy and dignity, and developing institutional accountability systems can contribute to reducing OV. Ultimately, we maintain that a substantial amount of work remains in comprehending and preventing OV, both in Türkiye and worldwide. Future research could address a significant gap by multidimensionally assessing the impact of education, monitoring, and infrastructure interventions developed to institutionalize respectful care in birth settings on women’s birth experiences, the attitudes and behaviors of birth workers, patient health indicators, and system-level quality improvement processes.

## Data Availability

All research data (voice recordings, transcripts and coded data) are stored. All data is recorded in our native language and can be shared if desired after publication. Data has not been shared with a public archive. The first author (kubraucdum@gmail.com) can be contacted for raw data.
